# Return rate following a live birth obtained with ART: frequency and determinants

**DOI:** 10.1007/s00404-024-07382-9

**Published:** 2024-02-04

**Authors:** Alessia Limena, Marco Reschini, Dalila Invernici, Fabio Parazzini, Letizia Li Piani, Paola Viganò, Edgardo Somigliana, Ludovica Basili

**Affiliations:** 1 Department of Clinical Sciences and Community Health, Università Degli Studi Di Milano, Milan, Italy; 2 Infertility Unit, Fondazione IRCCS Ca’ Granda Ospedale Maggiore Policlinico, Via M. Fanti, 6, 20122 Milan, Italy

**Keywords:** Second child, IVF, ART, Infertility, Demography

## Abstract

**Purpose:**

To understand how often couples return to ART centres for a second child.

**Methods:**

Retrospective monocentric cohort study including women who had a first live birth with IVF. The primary objective was to assess the rate of those returning for a second child within five years of the previous pregnancy. The secondary aim was to disentangle the determinants of this rate.

**Results:**

A total of 374 patients were included, of whom 188 returned (50%, 95% CI 45–55%). Among those who did not return (*n* = 186), four (2%) referred to another ART Center and 24 were unreachable. Of the 158 contacted subjects that did not refer for ART, 53 (34%, 95% CI 27–41%) conceived naturally, 57 (36%, 95% CI 29–44%) abandoned their intent of parenthood, and 48 (30%, 95% CI 24–38%) unsuccessfully attempted natural conception. These 48 women (13%) who expressed interest in a second child but did not undergo ART were compared to those seeking a second pregnancy through ART. Baseline characteristics were similar except for an older age (Median 36, IQR: 34–38 vs 34, IQR: 32–36, *p* = 0.001). Additionally, in terms of IVF cycle characteristics, women who did not return were more likely to achieve their first pregnancy with a fresh transfer rather than a frozen transfer (75% vs 59%, *p* = 0.05). They also had a higher number of retrieved oocytes (Median 10, IQR: 7–13 vs 9, IQR: 5–12) and less frequently cryopreserved embryos (27% vs 52%, *p* = 0.003).

**Conclusion:**

The proportion of couples who have conceived with ART and who are interested in having a second child is high. Our results underline the importance of paying more attention to the number of intended children, as this information could influence clinical management.

## What does this study add to the clinical work

**Table:** 

Most women conceiving their first child through ART are interested in having a second child. Our results strengthen the importance of improving the management of infertile couples by paying more attention to the number of intended children.	

## Introduction

In most affluent countries the total fertility rate (TFR) is below the replacement threshold of 2.1. In Western Europe, it is estimated to be 1.59 [1]. Despite a wide literature on the demographic trends of parity in the general population [2], this information is scarce for the group of infertile couples requiring assisted reproductive technology (ART). The intended number of children in this population, as well as the return rate of those who achieve a first live birth through ART, has been poorly explored. We identified only one study that specifically investigated the return rate for second ART-conceived children [3]. Analysing data from the Australian and New Zealand Assisted Reproductive Database, the authors reported an overall return rate of 43% and identified the main predictive factors for return to be a younger age and having been nulliparous prior to the pregnancy obtained with ART. The study did not provide information on the reasons for non-return, such as the rate of women conceiving other children naturally.

Improving our knowledge of the demographic characteristics and intentions of infertile women who need ART to conceive is important for at least two reasons: first, if the return rate is lower than in the general population, the barriers and how to overcome them would need to be investigated; second if, conversely, infertile women who start ART often return for further children, different management strategies could be considered, such as embryos (or oocytes) accumulation before embryo transfers are initiated. This could improve the chance of success when the woman returns for a second or third child, two or more years later, when the effectiveness is generally lower because of aging.

To shed more light on this issue, we retrospectively selected women who had a live birth with ART cycles in our Center and investigated the return rate for a second child, as well as the determinants of their decisions.

## Methods

The primary objective of this retrospective monocentric cohort study was to estimate the frequency of couples who, after obtaining a live birth at our Center, return for a second child within five years of the previous pregnancy. The secondary aim was to understand the reasons that influences this decision and identify the clinical determinants of non-referral for those who aimed at a second child but did not attend an ART center. The study was conducted at Fondazione IRCSS Ca’ Granda Ospedale Maggiore Policlinico, Milan, Italy. All women under 40 years of age who had obtained a singleton pregnancy and a live-born child through ART at our Infertility Unit between 2013 and 2015 (thus giving birth by the end of 2016) were selected. We excluded women who were not nulliparous at the first attempt and those with legal proceedings against our Center. The study was approved by the local ethics committee (Milano Area 2 382_2020). Informed consent was not requested because this was a retrospective study. However, all women referring to our Unit give an informed consent for their data to be used for research purposes and those who refused were excluded. In addition, verbal consent was obtained for those who were contacted by phone.

Women were initially selected using the software Meditex (Regensburg, Germany). For women who returned to our Center to have another child, we collected data on the previous IVF cycle leading to pregnancy and other treatments from the medical records. Patients who did not return to our Center were contacted by phone and were interviewed using a standardized questionnaire to find out whether they had conceived naturally in the meantime or whether they had referred to other Centers. We investigated the reasons for discontinuation among those who had not tried to become pregnant. The phone contacts were made by medical staff with expertise in ART who were qualified to answer patients’ questions. In the case of non-response, telephone calls could be repeated up to three times, usually changing the time and day of the week. Contacts were done between September 2021 and March 2022.

Based on the primary objective, if the population of couples conceiving with ART reflects the general local population, we expected approximately 53% to return for a second child [4]. Of these, 75% should have proceeded within five years. Globally, if women conceiving with ART behave as the general local population, one had to expect a rate of return of about 40% (0.53 × 0.75). Setting type I and II errors at 0.05 and 0.20, respectively, and aiming for an amplitude of the 95%CI of the proportion below 10%, the required sample size was about 350 women. We estimated that by retrospectively including women who conceived between 2013 and 2015 and gave birth by the end of 2016, we could achieve this sample size.

We used the software Statistical Package for Social Sciences (SPSS, Chicago, IL), version 27.0, to analyze the data. A binomial distribution model was used to assess the 95%CI of the most relevant proportions. For the comparisons between groups, Student’s t-test, Wilcoxon’s non-parametric test, Chi-squared test or Fisher’s exact test were used, depending on the type of variable. P values below 0.05 were considered statistically significant. In the analysis to identify predictive factors of return, variables found to significantly differ in the univariate analysis were entered into a multivariate logistic regression model to assess the adjusted Odds Ratio (OR) of the association.

## Results

**Fig. 1:**
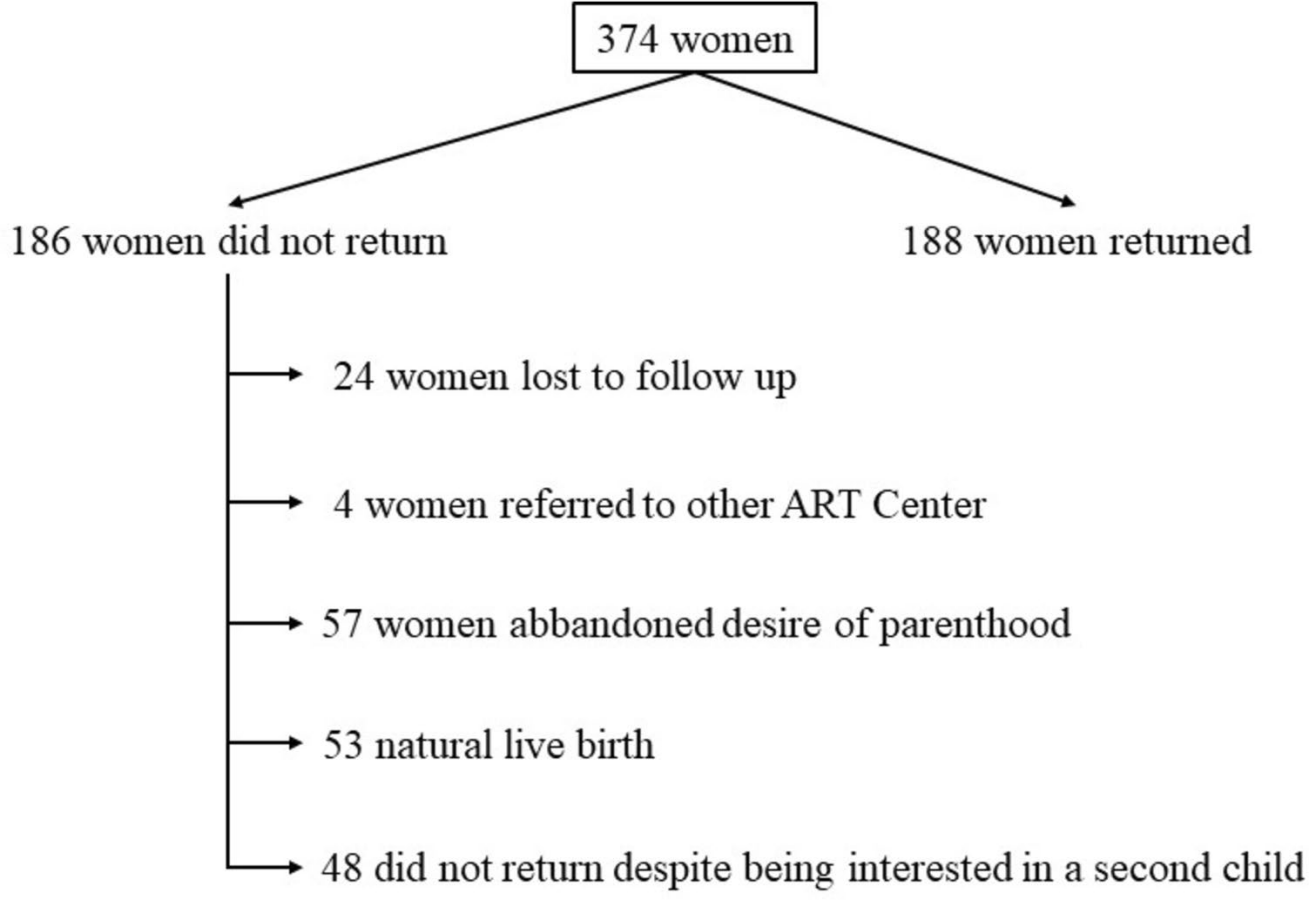
Flow Chart of the study

**Fig. 2:**
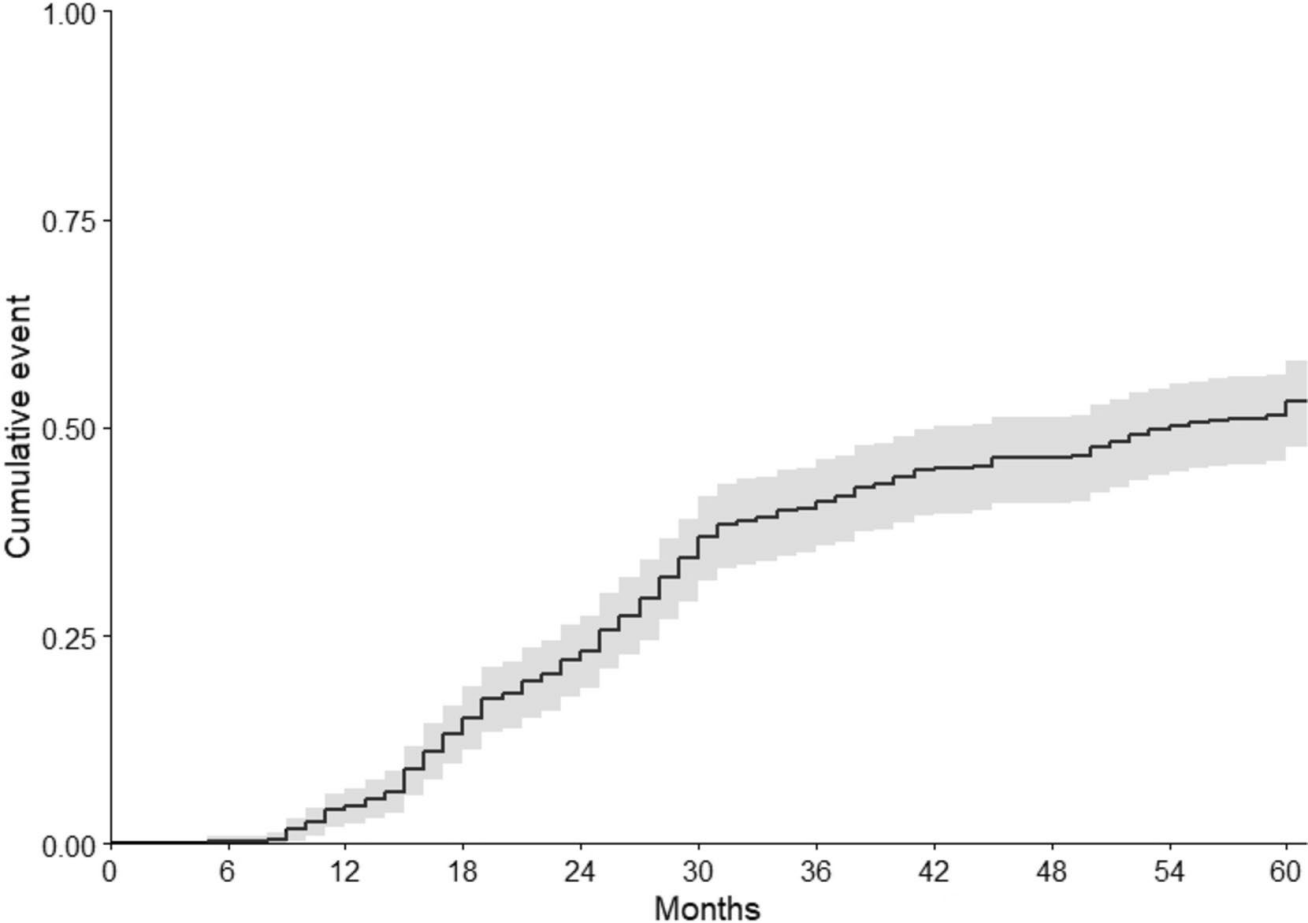
Survival analysis on the rate of return. The cumulative rate of return (95%CI) at 1, 2, 3, 4 and 5 years was 5% (2–7%), 23% (19–27%), 41% (36–46%), 47% (41–52%), and 53% (48–58%), respectively

We identified 374 patients who obtained pregnancy through ART at our Center between 2013 and 2015 and who gave birth within 2016. The cohort flowchart is summarized in Fig. 1. One hundred and eighty-eight of the 374 women referred to our Center within 5 years of delivery to attempt a second pregnancy (50%, 95% CI 45–55%). On average, patients returned to seek a second pregnancy after a median [IQR] of 2.2 [1.4–2.8] years. The survival curve is shown in Fig. 2. The cumulative rate of return (95%CI) at 1, 2, 3, 4 and 5 years was 5% (2–7%), 23% (19–27%), 41% (36–46%), 47% (41–52%), and 53% (48–58%), respectively. Of the 188 women who returned to our unit for further treatment, 106 (57%, 95%CI) had a live birth.

The 186 who did not return (374–188) were contacted by phone: 24 (13%) of them never replied and were therefore excluded from the analysis of reasons for non-return. In fact, we could not know whether they had conceived naturally, had new cycles at other centers or did not want more children. In addition, four referred to other ART Centers. Of the 158 contacted subjects that did not refer for ART, 53 conceived naturally (34%, 95% CI 27–41%), 57 gave up on parenthood (36%, 95% CI 29–44%), and 48 tried unsuccessfully to conceive naturally (30%, 95% CI 24–38%). Table 1 shows the specific reasons for non-return in the latter two groups. For the analysis of the clinical determinants of non-return, we focused on the 48 women who did not undergo ART despite being interested in having a second child. Overall, these women represented 13% (95% CI 10–17%) of the total cohort. The baseline characteristics of these 48 subjects were compared with those who sought a second pregnancy through ART either at our Center or elsewhere (188 + 4 = 192 subjects). The results of this analysis are shown in Table 2. Studied baseline clinical variables did not differ except for age. Women who returned were younger. Some differences emerged when focusing on the characteristics of the IVF cycle. Women who did not return were more likely to achieve their first pregnancy in a fresh cycle, to retrieve more oocytes, and to have fewer remaining frozen embryos. We set up a multivariate logistic regression model with the four variables found to significantly differ in the univariate analysis (age, first live birth achieved in a fresh cycle, number of oocytes retrieved and availability of remaining frozen embryos): only the availability of supernumerary frozen embryos remained significantly associated with the return rate, the adjusted OR being 2.07 (95% CI 1.31–3.27, *p* = 0.002).

**Table 1:** Women who have not returned

Main group of non-return and reasons	n
Abandoned desire of parenthood (*n* = 57)	
No longer desired children	19
Divorced	10
Health problems	12^a^
Challenging first child	11
Will seek a pregnancy in the future	3
Male partners died	2
Did not return despite being interested in a second child (*n* = 48)	
Too emotionally and physically stressful	13
Low probability of pregnancy	8
Natural conception	5^b^
Too challenging organizationally	1
Did not report patent reasons	14
Feared for her health	3
Other reasons	4

^a^Two women undergone an hysterectomy, one had breast cancer, eight advised against another pregnancy, one Multiple Sclerosis

^b^Four miscarriages and one fetal intrauterine death

**Table 2:** Baseline characteristics of the two study groups

Characteristics	Returned women	Non returned women	*p*
	*n* = 192	*n* = 48	
Age (years)	34 [32–36]	36 [34–38]	< 0.001
Nationality			0.15
Italian	148 (77%)	34 (79%)	
Western Europe	12 (6%)	6 (13%)	
Extra Europe	32 (17%)	4 (8%)	
BMI (Kg/m2)	21.8 [19.7–24.2]	21.1 [19.0–22.8]	0.07
AMH (ng/ml)	2.2 [1.2–3.6]	1.8 [0.9–4]	0.50
AFC	13 [9–8]	12 [7–16]	0.14
Duration of infertility (years)	3 [2–5]	4 [3–5]	0.42
LPS/LPT	44 (23%)	9 (19%)	0.70
Hysteroscopy	19 (10%)	2 (4%)	0.27
Previous pregnancies	40 (21%)	11 (23%)	0.84
Previous pick up			0.15
0	94 (49%)	16 (33%)	
1	66 (34%)	18 (38%)	
2	24 (13%)	10 (21%)	
≥ 3	8 (4%)	4 (8%)	
Previous ET			0.60
0	81 (42%)	18 (38%)	
1	60 (31%)	16 (33%)	
2	31 (16%)	2 (12%)	
≥ 3	20 (11%)	8 (17%)	
First pregnancy cicle			0.05
Fresh	113 (59%)	36 (75%)	
Crio	79 (41%)	12 (25%)	
Indication to IVF			0.08
Unexplained	35 (18%)	16 (33%)	
Tubal factor	28 (15%)	4 (8%)	
Ovulatory disorder	15 (8%)	0 (0%)	
Endometriosis	20 (10%)	7 (15%)	
Male	73 (38%)	16 (33%)	
Mixed	21 (11%)	5 (10%)	
Total oocytes	9 [5–12]	10 [7–13]	0.05
Residual Embryo cryo	99 (52%)	13 (27%)	0.003
Residual Ovo cryo	10 (5%)	0 (0%)	0.22
Underwight children (< 2500 gr)	13 (7%)	5 (10%)	0.37
Children with medical problems	2 (1%)	2 (4%)	0.18

Data are reported as median [interquartile range] or number (percentage)

## Discussion

Our study showed that one in two women who had a live birth with ART returned for new treatment cycles (50%, 95% CI 45–55%). This proportion is similar to, if not higher than, the expected rate of 40% based on available data for the local general population. In addition, our analysis showed that the survival curve tends to reach a plateau after 3–4 years, suggesting that prolonging follow-up will not significantly increase this rate. The high interest in second children among the infertile population is further supported by the observation that natural conception explained a significant proportion of women who did not return (34%, 95% CI 27–41%). Finally, our analysis highlighted that the main determinant of return is the availability of frozen embryos.

The return rate observed in our analysis is very similar to that reported by Paul et al. who showed an overall rate of 43% and a cumulative rate at 5 years of 50% [3]. However, the determinants of return were different. Younger age, a greater number of oocytes retrieved, being nulliparous prior to the first pregnancy achieved with ART, the use of ICSI rather than conventional IVF, a fresh rather than a frozen embryo transfer and the transfer of a single blastocyst were predictive factors of return in that previous study [3]. None of these factors were found to be significantly associated with return in our analysis. Differences in the studied population, local clinical practices of the ART Centres and, most importantly, study power could explain this difference. It should be noted that, although statistically significant, most of the variables identified by Paul et al. showed an association below 1.50 (or above 0.67), which calls into question the clinical relevance of the associations found. Only age and parity showed stronger associations. On the other hand, Paul et al. did not test the availability of remaining frozen blastocysts, which was the only variable associated with return in our analysis. In our opinion, this finding is of clinical interest, not only because of the magnitude of the association (OR = 2.07, 95% CI 1.31–3.27), but also because it may suggest a different clinical approach. Indeed, given the overall high rate of women interested in more than one child, one could foresee to perform more than one stimulation and accumulate frozen embryos (or frozen oocytes) prior to initiate the embryo transfers. This strategy may increase the chances of achieving the desired number of children. Of course, it cannot be recommended for everyone, but some women may benefit from it. These include those with an insurmountable obstacle to conception (whose chances to conceive naturally after childbirth are nil or close to nil) and those over the age of 35, as their chances of success may be significantly reduced if they return for a second child some years later [5]. To note, in our experience, 42% of women failed when they returned. For these women who did not achieve a second child with ART, the non-performance of an accumulation of embryos or oocytes prior to initiate the transfers could be seen as a lost opportunity. There is a consistent room for improvement here, given the better prognosis of women who have previously conceived with ART. The OR of ART success for women who already succeeded with ART was indeed shown to be 2.04 (95% CI 1.89–2.20) [6]. It may be important to actively consider the number of intended children at the time of ART initiation. IVF can be tailored to patients’ conditions and wishes. At present, however, there is still insufficient evidence to advocate a paradigm shift in ART management policy for all or selected couples. Further evidence is needed.

The higher return rate among those with frozen embryos deserves some additional considerations. First, it may reflect the parents’ cultural beliefs. They may idealize frozen embryos as unborn siblings and feel somehow obliged to return to give them their chance. Italian culture, characterized by a strong catholic commitment in favor of the embryos, may play a role here. If so, this could argue against the above-mentioned vision of a different ART policy with the accumulation of embryos prior to the initiation transfers. Couples should not feel obliged to come back for their embryos. Secondly, and not in contrast, the availability of frozen embryos may facilitate return because women do not have to face a new ovarian stimulation and oocyte retrieval. The emotional, psychological, physical, and economic burden of this phase of ART is overwhelming for many couples and has been recognized as an important cause of dropout. A meta-analysis on this topic showed that 18% of couples who fail the first attempt do not continue, a percentage that rises to 25% in couples who have failed two attempts [7]. Local data suggest that the scenario could be even worse in our context (29% dropout after the first failure and a further 42% after the second failure), even though ART is fully covered by the National Health System [8]. This latter finding further emphasizes that a 50% rate of return must be considered sizeable.

Some strengths and limitations of our study should be highlighted. Regarding the former, this is the first study to specifically address the return rate and its determinants by contacting patients directly. Paul et al. provided valuable and robust information on the return rate and its determinants, but, as they used regional health care utilisation databases, they could not provide any information on the reasons for non-return. Limitations include the relatively small sample size, the monocentric setting, which may prevent the generalizability of the data and the retrospective design that prevented the collection of more detailed clinical information.

In conclusion, the proportion of couples who conceived with ART and are interested in having a second child is high. If confirmed in other contexts, this finding could open to new scenarios for ART. The outcome of these techniques should shift from live birth to achieving the desired number of children. In addition, the procedure itself may change, allowing for the accumulation of embryos or oocytes before the start of the transfers, at least in the subgroup of women who may benefit more. However, more precise information on the rate of return and its determinants, as well as robust cost-effectiveness analyses, are needed before advocating such changes. Larger studies from other independent contexts are required.

## Data Availability

The data presented in this study are available on request from the corresponding author. The data are not publicly available due to privacy.
